# 2R,3R-trans-Dihydroquercetin Has Powerful Antioxidant Properties, Prevents DNA and Protein Damage, and Protects Mice from Injury Caused by Radiation-Induced Oxidative Stress

**DOI:** 10.3390/antiox15040423

**Published:** 2026-03-27

**Authors:** Olga Shelkovskaia, Anatoly V. Chernikov, Dmitriy A. Serov, Dmitriy E. Burmistrov, Yuri A. Trutnev, Ruslan M. Sarimov, Alexander V. Simakin, Eugeny M. Konchekov, Serazhutdin A. Abdullaev, Ekaterina E. Karmanova, Mars G. Sharapov, Sergey V. Gudkov

**Affiliations:** 1 BIOCAD, 142380 Lyubuchany, Russia; shelkovskayaov@biocad.ru; 2 Institute of Theoretical and Experimental Biophysics, Russian Academy of Sciences, 3 Institutskaya St., 142290 Pushchino, Russia; chernikovav@iteb.pushchino.ru; 3 Prokhorov General Physics Institute of the Russian Academy of Sciences, Vavilov Str. 38, 119991 Moscow, Russia; dmitriy_serov_91@mail.ru (D.A.S.); dmitriiburmistroff@gmail.com (D.E.B.); trutnev@kapella.gpi.ru (Y.A.T.); rusa@kapella.gpi.ru (R.M.S.); avsimakin@gmail.com (A.V.S.); eukmek@gmail.com (E.M.K.); 4 Burnasyan Federal Medical Biophysical Center, Federal Medical-Biological Agency of Russia, 46 Zhivopisnaya St., 123098 Moscow, Russia; sabdullaev@fmbcfmba.ru; 5 Federal Research Center Pushchino Scientific Center for Biological Research, Institute of Cell Biophysics, Russian Academy of Sciences, 3 Institutskaya St., 142290 Pushchino, Russia; karee@pbcras.ru (E.E.K.); sharapov-mg@pbcras.ru (M.G.S.); 6 Department of Fundamental Sciences, Bauman Moscow State Technical University, 5 2nd Baumanskaya St., 105005 Moscow, Russia

**Keywords:** 2R,3R-trans-dihydroquercetin, radioprotector, 8-oxoguanine, genes expression, leukopenia, thrombopenia

## Abstract

(1) Background: The search for new polymodal antioxidants to correct oxidative stress of various origins and its consequences remains one of the most pressing and rapidly developing areas of biomedical research. (2) Methods: Hydrogen peroxide and hydroxyl radical detection, induced luminescence assay, ELISA for 8-oxoguanine detection, animal survival, blood cell count, micronucleus test, and PCR were used. (3) Results: 2R,3R-trans-dihydroquercetin (DHQ) was shown to reduce the amount of hydrogen peroxide and hydroxyl radicals formed during water radiolysis, leading to reduced damage to biomolecules. DHQ is a radioprotector, most effective at a dose of 300 mg/kg administered 15 min before radiation exposure. The dose reduction factor is 1.22. DHQ administration reduces the severity of radiation-induced leukopenia and thrombopenia by protecting red bone marrow cells. The mechanism of DHQ’s radioprotective action is fundamentally different from that of classical stress response inducers and is based on the normalization of the target cell transcriptional profile, rather than its hyperstimulation. (4) Conclusions: DHQ’s ability to restore the expression of antioxidant defense, DNA repair, and apoptotic genes to physiological levels under radiation exposure allows it to be considered a promising pharmacological agent for the correction of radiation-induced damage to normal tissues.

## 1. Introduction

The search for new polymodal antioxidants to correct oxidative stress of various origins and its consequences remains one of the most pressing and rapidly developing areas of biomedical research [1]. A particular, yet socially and clinically significant, area of this problem is the development of new radioprotectors—molecular agents of various chemical natures capable of reducing the damaging effects of ionizing radiation [2]. The need for effective radioprotectors arose simultaneously with the development of nuclear technologies and remains relevant in the context of the expanding use of ionizing radiation in medicine, industry, and the energy sector [3].

A significant portion of the biological damage induced by widely used sources of ionizing radiation occurs through water radiolysis and the subsequent formation of free radicals, primarily reactive oxygen species (ROS), in the body’s aquatic environments [4]. The resulting ROS initiate cascade reactions of lipid peroxidation, damage to proteins and nucleic acids, leading to the development of acute oxidative stress and disruption of cellular homeostasis [5]. Therefore, the development of radioprotectors has traditionally focused primarily on compounds with pronounced antioxidant properties, including free radical scavengers and ROS scavengers [6]. Such drugs are typically administered shortly before irradiation and are aimed at reducing the severity of acute radiation-induced oxidative stress [7].

Potential radioprotectors must meet strict requirements: chemical and metabolic stability, ease and safety of administration, low toxicity at therapeutically effective doses, and minimal impact on the antitumor efficacy of radiation therapy [8,9,10]. Currently, a significant number of compounds with radioprotective activity have been described, some of which have been approved for clinical use. These include thiol-containing drugs (amifostine (WR-2721) and its active thiol metabolite WR-1065), phytochemicals, vitamins (ascorbic acid, α-tocopherol, etc.), hormones and their synthetic analogs (melatonin, indralin), and other substances. However, their clinical use is often limited by severe side effects, a narrow therapeutic window, or insufficient efficacy [6]. Therefore, the search for and development of safe and highly effective radioprotective drugs remains a pressing issue in modern radiobiology and pharmacology.

Dihydroquercetin (DHQ, taxifolin, 2-(3,4-dihydroxyphenyl)-3,5,7-trihydroxy-2,3-dihydro-4H-1-benzopyran-4-one) is a naturally occurring compound that belongs to the flavanol subclass of the flavonoid family. DHQ has a significant range of biological effects, including antioxidant, antibacterial, antiviral, anti-inflammatory, and antiapoptotic activities. In experimental studies, DHQ has demonstrated hepatoprotective, antidiabetic, cardioprotective, anticancer, and neuroprotective effects [11]. For example, DHQ effectively reduces oxidative stress in vitro and in vivo during hypothermia [12].

**Figure 1:**
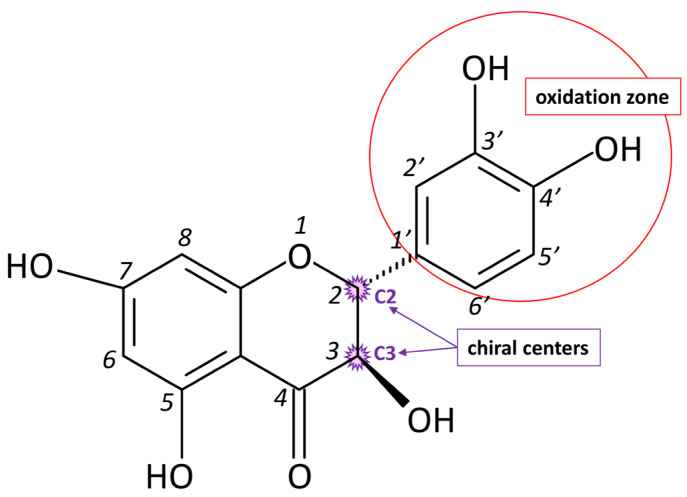
Structure of DHQ. Chiral centers are marked with asterisks.

The main limitations and disadvantages of DHQ include low bioavailability, low stability, and rapid metabolism, which reduce its therapeutic efficacy. However, this problem is being successfully addressed through nanotechnology and other developments [11]. An additional aspect requiring in-depth study is the stereochemical heterogeneity of DHQ. The molecule contains two chiral centers (C2 and C3) and, accordingly, can exist as four stereoisomers (Figure 1). The main natural and most biologically active one is 2R,3R-trans-dihydroquercetin. In addition, the 2S,3S-trans-, as well as 2R,3S- and 2S,3R-cis-forms, have been described [13]. It was previously noted that the antioxidant activity of DHQ depends on the enantiomeric composition of the drug [14], which indicates the fundamental role of spatial configuration in the implementation of its biological effects. This phenomenon is also observed for other drugs, for example, for α-lipoic acid, which also has antioxidant and radioprotective properties [15]. In this regard, the aim of the present work was to study the antioxidant and radioprotective activity of the 2R,3R-trans-dihydroquercetin isomer as the most promising form of the compound from a pharmacological point of view.

## 2. Materials and Methods

### 2.1. Exposure to Ionizing Radiation

Irradiation was performed using a RUT-15 X-ray therapeutic system (15 mA, 200 kV) (Moscow, Russia) at a dose rate of 1 Gy/min and a focal length of 37.5 cm. Salmon sperm DNA solutions (350 μg/mL in 1 mM phosphate buffer, pH 6.8) were irradiated in siliconized Eppendorf tubes (Oldenburg, Germany) (500 μL). Water, aqueous solutions of coumarin-3-carboxylic acid, and aqueous protein colloids were irradiated in glass scintillation vials. Total irradiation of the animals was performed in a special cylindrical cage, which restrained their vertical movement.

### 2.2. Hydrogen Peroxide Concentration Determination

To quantify hydrogen peroxide concentration in aqueous solutions, a highly sensitive enhanced chemiluminescence method using a luminol-p-iodophenol-horseradish peroxidase system was used. Experiments were conducted on a Biotox-7A chemiluminometer (Moscow, Russia) (Russia). The concentration of hydrogen peroxide formed was calculated using calibration curves, which were constructed by measuring the chemiluminescence intensity of samples containing added hydrogen peroxide of known concentration. The initial H_2_O_2_ concentration used for calibration was determined spectrophotometrically at a wavelength of 240 nm with a molar absorption coefficient of 43.6 (M^−1^ × cm^−1^). Before measurement, samples were placed in polypropylene vials (Beckman, Brea, CA, USA), and 1 mL of a “counting solution” containing 1 cM Tris-HCl buffer pH 8.5, 50 μM p-iodophenol, 50 μM luminol, and 10 nM horseradish peroxidase was added. The “counting solution” was prepared immediately before measurement. The sensitivity of the method allows the determination of H_2_O_2_ at a concentration of <1 nM [16].

### 2.3. Determination of OH-Radicals

This was carried out using a reaction with coumarin-3-carboxylic acid (CCA), the hydroxylation product of which, 7-hydroxycoumarin-3-carboxylic acid (7-OH-CCA), is a convenient fluorescent probe for determining the formation of these radicals [16]. A 0.2 M phosphate buffer (pH 6.8) was added to a CCA solution in water (0.5 mM, pH 3.6), followed by DHQ. The test samples (and controls) were exposed to X-ray irradiation (Moscow, Russia). The fluorescence of the reaction product of CCA with the hydroxyl radical, 7-OH-CCA, was measured on a Jasco 8300 spectrofluorimeter (Jasco, Tokyo, Japan) with λ_ex_ = 400 nm, λ_em_ = 450 nm. Calibration was performed using commercial 7-OH-CCA.

### 2.4. Determination of 8-Oxoguanine Content in DNA

To quantify 8-oxoguanine in DNA, a noncompetitive enzyme-linked immunosorbent assay using monoclonal antibodies specific for 8-oxoguanine was developed [17]. After irradiation, DNA samples (350 μg/mL) were denatured by boiling in a water bath for 5 min and cooled on ice for 3–4 min. Aliquots (42 μL) were applied to the bottom of wells of enzyme-linked immunosorbent plates. DNA was immobilized for 3 h at 80 °C until the solution was completely dry. Nonspecific adsorption sites were blocked using 300 μL of a solution containing 1% dry skim milk in 0.15 M Tris-HCl buffer, pH 8.7, and 0.15 M NaCl. The plates were then incubated at room temperature overnight (14–18 h). The formation of the antigen–antibody complex (100 μL/well) with antibodies specific to 8-OG (diluted 1:2000) was carried out in a blocking solution by incubation for 3 h at 37 °C. The mixture was washed twice (300 μL/well) with a solution of 50 mM Tris-HCl buffer, pH 8.7, and 0.15 M NaCl with 0.1% Triton X-100 after 20 min of incubation. Then, a complex was formed with the conjugate (anti-mouse immunoglobulin labeled with horseradish peroxidase (1:1000) in a blocking solution (80 μL/well), incubating for 1.5 h at 37 °C. Then, the wells were washed 3 times in the same way as described above. Next, a chromogenic substrate was added: 18.2 mM ABTS and hydrogen peroxide (2.6 mM) in 75 mM citrate buffer, pH 4.2 (100 μL/well). Upon achieving coloration, the reaction was stopped by adding an equal volume of 1.5 mM NaN_3_ in 0.1 M citrate buffer, pH 4.3. The optical density of the samples was measured at λ = 405 nm.

### 2.5. Measuring the Induced Luminescence of Protein Solutions

The amount of long-lived reactive protein species formed after exposure to ionizing radiation was estimated based on the luminescence intensity of aqueous protein colloids [18]. The induced luminescence of protein solutions was measured at different times after exposure to X-rays using Biotox 7A chemiluminometer (Moscow, Russia). All BSA solutions were prepared in 20 mM Tris-HCl buffer, pH 8.0. Measurements were performed in potassium-free glass vials. Chemiluminescence of the samples was measured for 30 s.

### 2.6. Survival Test

Males of randombred white Kv:SHK mice (Kryukovo nursery (Kryukovo, Russia), Russian Academy of Medical Sciences) were used in the experiments. The animals were kept in a vivarium and fed a standard diet with free access to pelleted commercial mouse chow (Arno, Moscow, Russia) and tap water. The institutional guide for the care and use of laboratory animals was carefully followed. All experimental protocols received approval from the Bioethics Committee of the Institute of Theoretical and Experimental Biophysics, Russian Academy of Sciences. Male Kv:SHK mice weighing 28–31 g and aged 10 weeks were tested for survival. Mice were injected intraperitoneally with DHQ dissolved in 0.14 M sodium chloride solution. The injection was 0.4 mL per mouse. Subsequently, deaths were counted once per day. Two groups of mice injected with isotonic saline, irradiated at the same dose or not exposed to irradiation, served as controls. In some experiments, mice were weighed daily, and food and water consumption was recorded. Necropsy was performed as described in [19].

### 2.7. Micronucleus Assay

Cytogenetic damage to cells was assessed by the appearance of polychromatophilic erythrocytes (PCEs) containing micronuclei (MNs). Mice were sacrificed by cervical dislocation 24 h after irradiation with dose of 1 Gy, as the maximum release of PCE containing MNs is observed approximately 24 h after exposure to ionizing radiation. Histological preparations were prepared and stained using the standard method with modified cell dissolution [20]. PCEs containing MNs were counted using a MikMed-2 light microscope (LOMO, Saint Petersburg, Russia) with an immersion objective at ×1000 magnification. Data were analyzed for 5 individuals per experimental point, with a minimum of 2000 PCEs counted per mouse.

### 2.8. Blood Count

Five mice, randomly selected from a group of 10 animals, had their peripheral blood counts calculated, and the results were then averaged across these five animals. At the end of the experiment, if fewer than five mice survived in the group, blood was collected from all remaining animals. Peripheral blood samples were taken from the tail vein. All experimental procedures have been described in detail previously [19].

### 2.9. Gene Expression

Real-time PCR (RT-PCR) was used to analyze gene expression in mouse red bone marrow cells after exposure to (ionizing) X-rays and administration of exogenous DHQ. Bone marrow cells were isolated from the excised proximal femoral epiphysis using an insulin syringe and resuspended in 100 μL of 10% fetal serum. Total RNA from the bone marrow cells was isolated using ExtractRNA reagent (Eurogen, Moscow, Russia). RNA quality was assessed by electrophoresis in 2% agarose gel in TAE buffer, in the presence of ethidium bromide (1 μg/mL). RNA concentration was determined using a NanoDrop 1000c spectrophotometer (Wilmington, DL, USA). To remove possible genomic DNA contamination, the obtained RNA was treated with DNase RQ1 (Promega, Madison, WI, USA). For reverse transcription (RT), 2 μg of total RNA and the MMLV RT kit (Eurogen, Moscow, Russia) were used. cDNA obtained as a result of reverse transcription was used in PCR with gene-specific primers (Table 1) manufactured by Evrogen (Moscow, Russia). Real-time PCR was performed in a BioRad CFX96 amplifier (BioRad, Hercules, CA, USA) using the qPCRmix-HS kit (Eurogen, Moscow, Russia) containing SYBR Green II (Molecular Probes Inc., Waltham, MA, USA) as a fluorescent intercalating dye. The PCR regime was as follows: (1) “hot start” (to exclude non-specific annealing of primers) at 95 °C for 5 min, (2) denaturation at 95 °C for 15 sec, (3) primer annealing at 58–60 °C for 20 sec, and DNA synthesis at 72 °C for 20 sec. Steps 2–4 were repeated 40 times. The threshold cycle or Ct values were determined using BioRad software LightCycler 96 (BioRad, Hercules, CA, USA). Normalization was performed relative to the cytoskeletal actin gene (*Actb*). The results were calculated using the standard method [21]. ΔΔCt was calculated using the formula ΔΔCt = ΔCt (control) − ΔCt (experiment), and each ΔCt value was calculated using the formula ΔCt = ΔCt (antioxidant enzyme gene) − ΔCt (Actb reference gene).

## 3. Results

**Figure 2:**
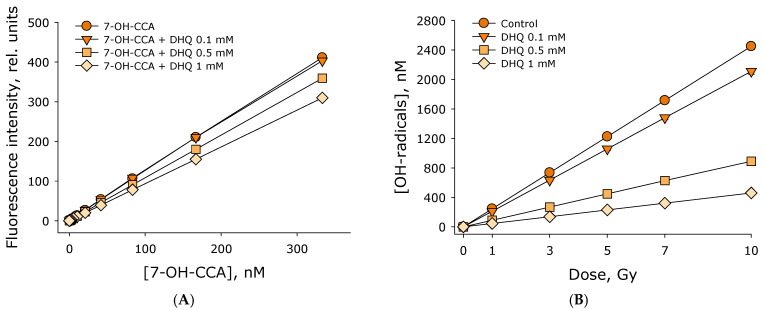
Effect of 2R,3R-trans-dihydroquercetin on the generation of hydroxyl radicals in water exposed to ionizing radiation. (**A**) Effect of 2R,3R-trans-dihydroquercetin on the fluorescence intensity of 7-OH-coumarin-3-carboxylic acid, the main fluorescent product of the reaction of coumarin-3-carboxylic acid and hydroxyl radicals. (**B**) Generation of hydroxyl radicals in water in the presence of different concentrations of 2R,3R-trans-dihydroquercetin exposed to ionizing radiation at doses of 1–10 Gy. Data are presented as mean values ± standard errors of the mean (*n* = 3). Standard errors of the mean are smaller than the size of the points.

The effect of 2R,3R-trans-dihydroquercetin on the generation of hydroxyl radicals in water exposed to ionizing radiation was studied. The amount of hydroxyl radicals was estimated using a fluorescent trap for hydroxyl radicals of coumarin-3-carboxylic acid, the hydroxylation product of which exhibits intense fluorescence. The effect of 2R,3R-trans-dihydroquercetin on the fluorescence intensity of 7-OH-coumarin-3-carboxylic acid was studied (Figure 2A). It was shown that the fluorescence intensity depends linearly on the concentration of 7-OH-coumarin-3-carboxylic acid up to a concentration of 350 nM in all studied cases. The addition of 2R,3R-trans-dihydroquercetin at a concentration of 0.1 mM does not significantly affect the fluorescence intensity. Addition of 0.5 mM 2R,3R-trans-dihydroquercetin reduces fluorescence intensity by slightly more than 10% relative to the control. Addition of 1 mM 2R,3R-trans-dihydroquercetin reduces fluorescence intensity by approximately 25% relative to the control. Thus, calibration curves relating fluorescence intensity to the concentration of 7-OH-coumarin-3-carboxylic acid were obtained using commercial 7-OH-coumarin-3-carboxylic acid.

The effect of different 2R,3R-trans-dihydroquercetin concentrations on hydroxyl radical generation in water exposed to ionizing radiation at doses of 1–10 Gy was studied (Figure 2B). The number of hydroxyl radicals formed was shown to be linearly dependent on the ionizing radiation dose in the range of 1–7 Gy. The radiation-chemical yield of hydroxyl radicals in water exposed to X-rays is approximately 245 nM/Gy. When 2R,3R-trans-dihydroquercetin is added at a concentration of 0.1 mM, the number of hydroxyl radicals formed also linearly depend on the dose of ionizing radiation. The radiation-chemical yield of hydroxyl radicals was approximately 212 nM/Gy, which is 13% lower than in water without DHQ (control). When 2R,3R-trans-dihydroquercetin was added at concentrations of 0.5 and 1 mM, the number of hydroxyl radicals formed also linearly depends on the dose of ionizing radiation. The radiation-chemical yield of hydroxyl radicals at a DHQ concentration of 0.5 mM was approximately 89 nM/Gy, while at a DHQ concentration of 1.0 mM it was 46 nM/Gy, which is 43% and 81% lower than the control. Thus, it was demonstrated that 2R,3R-trans-dihydroquercetin effectively reduces the amount of hydroxyl radicals during water radiolysis.

**Figure 3:**
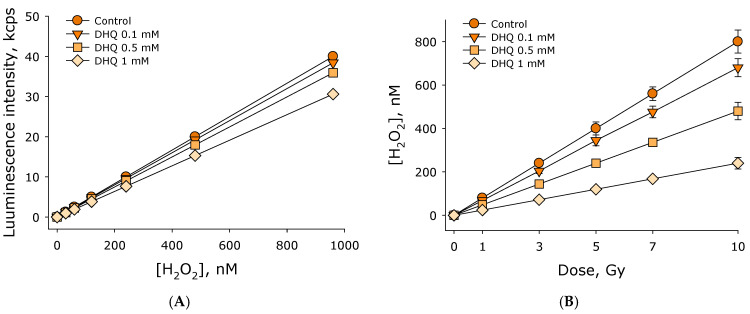
The effect of 2R,3R-trans-dihydroquercetin on the formation of hydrogen peroxide in water under the influence of ionizing radiation. (**A**) The effect of 2R,3R-trans-dihydroquercetin on the luminescence intensity of aqueous solutions containing different amounts of hydrogen peroxide. (**B**) Formation of hydrogen peroxide in water in the presence of different concentrations of 2R,3R-trans-dihydroquercetin under the influence of ionizing radiation at doses of 1–10 Gy. Data are presented as mean values ± standard errors of the mean (*n* = 3). Standard errors of the mean are less than the size of the points.

The effect of 2R,3R-trans-dihydroquercetin on the formation of hydrogen peroxide in water exposed to ionizing radiation was studied. The concentration of hydrogen peroxide was estimated using enhanced chemiluminescence by adding “counting solution” containing luminol-4-iodophenol-horseradish peroxidase. The effect of 2R,3R-trans-dihydroquercetin on the luminescence intensity of solutions with a known concentration of hydrogen peroxide was studied (Figure 3A). It was shown that the luminescence intensity depends linearly on the concentration of hydrogen peroxide up to a concentration of almost 1000 nM in all studied cases. The addition of 2R,3R-trans-dihydroquercetin at a concentration of 0.1 mM does not significantly affect the luminescence intensity. The addition of 0.5 mM 2R,3R-trans-dihydroquercetin reduces luminescence intensity by slightly more than 10% relative to the control. The addition of 1 mM 2R,3R-trans-dihydroquercetin reduces chemiluminescence intensity by approximately 25% relative to the control. Thus, calibration curves were obtained linking luminescence intensity and hydrogen peroxide concentration.

The effect of different 2R,3R-trans-dihydroquercetin concentrations on hydrogen peroxide formation in water exposed to ionizing radiation at doses of 1–10 Gy was studied (Figure 3B). It was shown that the amount of hydrogen peroxide formed linearly depends on the ionizing radiation dose in the range of 1–10 Gy. The radiation-chemical yield of hydrogen peroxide in water exposed to X-rays is approximately 80 ± 4 nM/Gy. When adding 2R,3R-trans-dihydroquercetin at a concentration of 0.1 mM, the amount of hydrogen peroxide formed also linearly depends on the dose of ionizing radiation. In this case, the radiation-chemical yield of hydrogen peroxide was approximately 68 nM/Gy, which is 15% less than in the control. When adding 2R,3R-trans-dihydroquercetin at concentrations of 0.5 and 1 mM, the amount of hydrogen peroxide formed also linearly depends on the dose of ionizing radiation. The radiation-chemical yield of hydrogen peroxide at a DHQ concentration of 0.5 mM was approximately 48 nM/Gy, and at a DHQ concentration of 1.0 it was mM–24 nM/Gy, which are 40% and 70% less than in the control. In a separate series of experiments, 1 mM DHQ was added to an aqueous solution of hydrogen peroxide at a concentration of 1 μM. Thus, it was demonstrated that 2R,3R-trans-dihydroquercetin effectively reduces the amount of hydrogen peroxide formed during water radiolysis.

**Figure 4:**
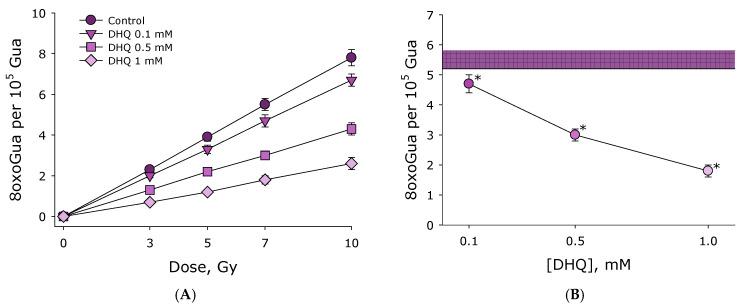
The effect of 2R,3R-trans-dihydroquercetin on the formation of 8-oxoguanine in DNA in vitro under the influence of ionizing radiation. (**A**) The effect of 2R,3R-trans-dihydroquercetin on the formation of 8-oxoguanine in DNA in vitro under the influence of ionizing radiation at dose of 3–10 Gy. (**B**) Formation of 8-oxoguanine in DNA in vitro under the influence of ionizing radiation at dose of 7 Gy in the presence of different concentrations of 2R,3R-trans-dihydroquercetin. The shaded area corresponds to the data obtained in the control group. Data are presented as mean values ± standard errors of the mean (*n* = 3). Background concentrations of 8-oxoguanine in DNA are subtracted from the presented results. Statistically significant differences between the control group and other groups are marked with asterisks.

The effect of 2R,3R-trans-dihydroquercetin on the formation of 8-oxoguanine in DNA in vitro under the influence of ionizing radiation was studied. The formation of 8-oxoguanine in DNA was assessed using an enzyme-linked immunosorbent assay using monoclonal antibodies to 8-oxoguanine. The effect of 2R,3R-trans-dihydroquercetin on the formation of 8-oxoguanine in DNA in vitro under the influence of ionizing radiation was studied (Figure 4A). It was shown that the concentration of 8-oxoguanine in DNA is linearly related to the dose of ionizing radiation in the range of 3–10 Gy. The data obtained in control experiments in the absence of DHQ can be linearized using the function *y* = 7.8*x* (R^2^ = 0.99), where y is the number of 8-oxoguanine bases in DNA per 100,000 guanine bases in DNA. *x* is the dose of ionizing radiation in Gy. In the presence of 0.1 mM DHQ, the data can be linearized using the function *y* = 6.7*x* (R^2^ = 0.99). In the presence of 0.5 mM DHQ, the data can be linearized using the function *y* = 4.3*x* (R^2^ = 0.99). In the presence of 1.0 mM DHQ, the data can be linearized using the function *y* = 2.6*x* (R^2^ = 0.97). Overall, the addition of 0.1 mM DHQ reduces the amount of 8-oxoguanine formed in DNA by slightly less than 15% (Figure 4B). With the addition of 0.5 mM DHQ, the amount of 8-oxoguanine formed is 45% less than in the control. With the addition of 1.0 mM DHQ, the amount of 8-oxoguanine formed is already 65% less than in the control. Thus, DHQ was shown to prevent radiation-mediated damage of DNA.

**Figure 5:**
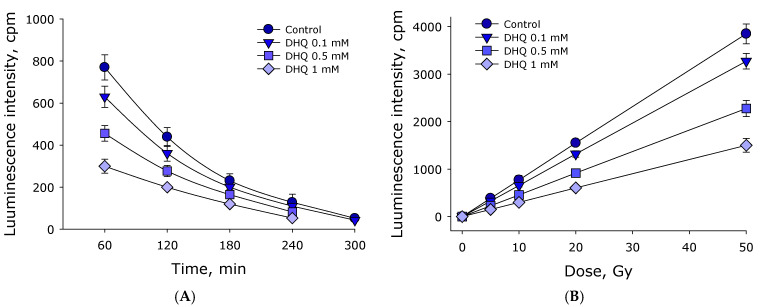
The effect of 2R,3R-trans-dihydroquercetin on the formation and elimination of long-lived reactive proteins species under the influence of ionizing radiation. (**A**) Dynamics of elimination of long-lived reactive proteins species in the presence of different concentrations of 2R,3R-trans-dihydroquercetin after exposure to ionizing radiation at dose of 10 Gy. (**B**) The effect of 2R,3R-trans-dihydroquercetin on the formation of long-lived reactive proteins species under the influence of ionizing radiation at dose of 5–50 Gy. Induced luminescence was measured after 1 h. Data are presented as mean values ± standard errors of the mean (*n* = 3). Baseline luminescence values are subtracted.

The effect of 2R,3R-trans-dihydroquercetin on the formation and elimination of long-lived reactive protein species exposed to ionizing radiation was studied using the induced luminescence method. The dynamics of the elimination of long-lived reactive protein species was determined in the presence of different concentrations of 2R,3R-trans-dihydroquercetin after exposure to ionizing radiation at a dose of 10 Gy (Figure 5A). In the first few tens of minutes after the end of exposure to ionizing radiation, extremely intense induced luminescence (2500–3000 cpm) is observed, which rapidly decreases. Conducting studies under conditions of rapidly decreasing signal is associated with various difficulties; therefore, signal recording was performed 1 h after irradiation. In the control, without the addition of DHQ, the induced luminescence intensity was shown to decrease by more than an order of magnitude over the course of the experiment (4 h). The half-life of long-lived reactive protein species was greater than 1.5 h. Adding DHQ at a concentration of 0.1 mM reduced the initial signal intensity by approximately 15%, while the half-life of long-lived reactive protein species remained unchanged. Adding DHQ at concentrations of 0.5 and 1.0 mM reduced the initial signal intensity by 40 and 60%, respectively. The half-life of long-lived reactive protein species became closer to 2 h.

The effect of 2R,3R-trans-dihydroquercetin on the formation of long-lived active forms of the protein was studied under exposure to ionizing radiation at doses of 5–50 Gy (Figure 5B). The rate of formation of long-lived reactive protein species was shown to be linearly dependent on the ionizing radiation dose. The induced luminescence intensity in the control was approximately 77 cpm/Gy. With the addition of DHQ at a concentration of 0.1 mM, the induced luminescence intensity was approximately 65 cpm/Gy, i.e., 15% lower than in the control. With the addition of DHQ at concentrations of 0.5 and 1.0 mM, the induced luminescence intensity was approximately 45 and 30 cpm/Gy, respectively, which was 40 and 60% lower than in the control. Thus, DHQ was shown to prevent radiation-mediated damage of proteins.

**Figure 6:**
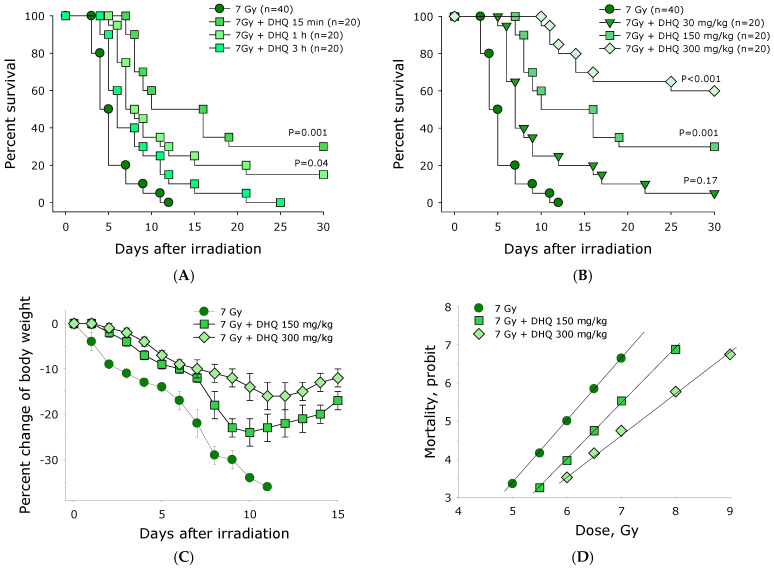
Effect of 2R,3R-trans-dihydroquercetin on animal survival after exposure to ionizing radiation. (**A**) Effect of the time of intraperitoneal administration of 2R,3R-trans-dihydroquercetin (150 mg/kg) before exposure to ionizing radiation at dose of 7 Gy on animal survival. Data are presented as the Kaplan–Meier plot. (**B**) Effect of the concentration of 2R,3R-trans-dihydroquercetin when administered intraperitoneally 15 min before exposure to ionizing radiation at dose of 7 Gy on animal survival. Data are presented as the Kaplan–Meier plot. (**C**) Effect of the concentration of 2R,3R-trans-dihydroquercetin on changes in animal weight after exposure to ionizing radiation at dose of 7 Gy. Data were obtained for 10 animals in each group. 2R,3R-trans-dihydroquercetin was administered intraperitoneally 15 min before radiation exposure. (**D**) Dependence of the probability of death on the dose of ionizing radiation. The number of animals in each group was at least 10. 2R,3R-trans-dihydroquercetin was administered intraperitoneally 15 min before radiation exposure. Data are presented as a probit plot.

The effect of 2R,3R-trans-dihydroquercetin on animal survival after exposure to ionizing radiation was studied. DHQ at a concentration of 150 mg/kg was shown to exhibit a protective effect when administered intraperitoneally to mice at different time intervals before exposure to ionizing radiation at a lethal dose of 7 Gy (Figure 6A). The average survival time of mice irradiated with dose of 7 Gy and not receiving DHQ was five days after irradiation, with the maximum survival time in this group being 12 days. Administration of DHQ at a concentration of 150 mg/kg significantly reduced the mortality of irradiated mice. DHQ was administered intraperitoneally to mice 15 min, 1 h, and 3 h before exposure to ionizing radiation. The survival rates of mice administered DHQ in this manner were 30%, 15%, and 0%, respectively. The median survival time of mice treated with DHQ in this manner was 16, 8, and 7 days, respectively. Thus, the most effective time is DHQ administration immediately before exposure to ionizing radiation (15 min).

The effect of different concentrations of 2R,3R-trans-dihydroquercetin administered intraperitoneally 15 min before exposure to 7 Gy of ionizing radiation on animal survival was studied (Figure 6B). Concentrations of 30, 150, and 300 mg/kg used in vivo were similar to concentrations of 0.1, 0.5, and 1.0 mM used in in vitro experiments. In the 7 Gy control group, the median survival was 5 days, and the maximum survival time was 12 days. When mice received DHQ at a concentration of 30 mg/kg 15 min before radiation exposure, the median survival was 7 days, with 5% of animals remaining alive by day 30 of the experiment. At a DHQ concentration of 150 mg/kg, the median survival was 16 days, with 30% of animals remaining alive by day 30 of the experiment. At a DHQ concentration of 300 mg/kg, 60% of animals remained alive by day 30 of the experiment. It is also important to note that in the control group of irradiated mice, the first lethal outcomes were recorded on the 4th day after the start of the experiment. In the group that received DHQ before irradiation, the first lethal outcomes were recorded significantly later. Thus, with increasing DHQ concentration, the effectiveness of radioprotection increases. Moreover, the concentration of 300 mg/kg is quite close to the solubility limit. It should be noted that we attempted to administer DHQ at a concentration of 300 mg/kg to non-irradiated mice. DHQ was shown to have no effect on the survival of non-irradiated animals.

In parallel with survival, the weight of mice was recorded. The weight of non-irradiated mice, both those receiving and those not receiving DHQ, increased by approximately 8% over the course of the experiment. After irradiation, mice not receiving DHQ lost weight throughout the experiment until complete death (Figure 6C). Thus, during the first day after exposure to 7 Gy of ionizing radiation, mice lost an average of 4% of their body weight. By day 11 of the experiment, a maximum weight loss of 36% was observed. After irradiation, mice receiving DHQ experienced significantly less weight loss. The maximum weight loss in the group of irradiated animals receiving DHQ at a concentration of 150 mg/kg was 24% by day 10. In the group of irradiated animals receiving DHQ at a concentration of 300 mg/kg, maximum weight loss was observed by day 12 and amounted to 16%. After passing the point of maximum weight loss, the animals began to gain weight.

The dose–response curve is convenient for calculating LD50/30 (the dose causing 50% lethality within 30 days). For control mice exposed to ionizing radiation and not receiving DHQ, the LD50/30 was 6 Gy (Figure 6D). Mice receiving DHQ at a concentration of 150 mg/kg had an LD50/30 of approximately 6.7 Gy. Mice receiving DHQ at a concentration of 300 mg/kg had an LD50/30 of approximately 7.3 Gy. The dose reduction factor (DRF), calculated as the ratio of the lethal doses in the presence and absence of DHQ, was 1.12 for a DHQ concentration of 150 mg/kg and 1.22 for a DHQ concentration of 300 mg/kg.

In all groups of animals irradiated at a dose of 7 Gy, no signs of diarrhea or overt rectal bleeding were observed. Postmortem examination also revealed no gastrointestinal tract distension, obstruction, intussusception, or hemorrhage. However, hemorrhages were observed in the lungs and pericardium of irradiated mice. Visually, the area of hemorrhages in the lungs of irradiated mice not receiving DHQ was larger than in the groups of irradiated mice receiving DHQ. Therefore, death of the mice was not due to gastrointestinal syndrome. The cause of death was likely related to damage to the hematopoietic system and the development of hematopoietic syndrome.

**Figure 7:**
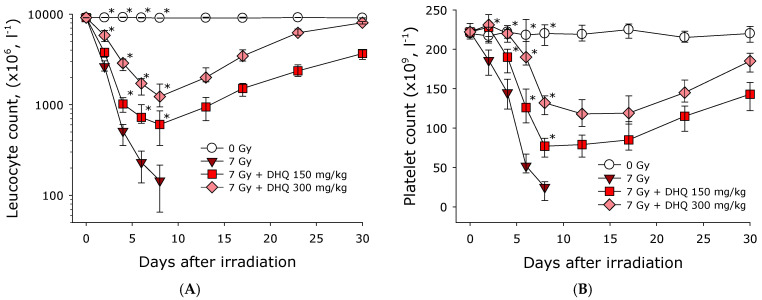
Effect of intraperitoneal administration of 2R,3R-trans-dihydroquercetin on the number of leukocytes (**A**) and platelets (**B**) in the bloodstream of mice exposed to ionizing radiation at dose of 7 Gy. Administration of 2R,3R-trans-dihydroquercetin was performed 15 min before irradiation. Data are presented as median, lower and upper quartiles. Statistically significant differences between the 7 Gy group and other groups are marked with asterisks (Mann–Whitney test, *p* < 0.05).

The effect of a single intraperitoneal administration of 2R,3R-trans-dihydroquercetin at concentrations of 150 and 300 mg/kg on the number of leukocytes in the peripheral blood of mice, both exposed and not exposed to ionizing radiation at a dose of 7 Gy, was studied (Figure 7A). In the group of non-irradiated animals (0 Gy), the number of leukocytes in the bloodstream did not change significantly during the experiment. Administration of DHQ to intact mice also did not cause any noticeable changes in the number of leukocytes in the bloodstream. After exposure to ionizing radiation, a decrease in the number of leukocytes in the bloodstream was observed in all groups of animals for 7 days. The number of leukocytes in mice irradiated at a dose of 7 Gy, which did not receive DHQ, decreased by 98% compared to the norm. This low white blood cell count was observed in this group of animals until their death. In the group of irradiated mice receiving DHQ at a concentration of 150 mg/kg, the white blood cell count in the peripheral blood decreased by 65% by day 7. By the end of the experiment, the white blood cell count in this group differed from the norm by approximately 35%. In the group of irradiated mice receiving DHQ at a concentration of 300 mg/kg, the white blood cell count in the peripheral blood decreased by 40% by day 7. By the end of the experiment, the white blood cell count in this group differed from the norm by approximately 15%. The effect of intraperitoneal administration of DHQ 15 min before exposure to ionizing radiation on the number of granulocytes in the peripheral blood of mice was studied. Changes in the number of granulocytes in the blood of all studied groups were similar to the changes observed in leukocytes. Thus, administration of DHQ significantly reduces the severity of radiation-induced leukopenia.

The effect of single intraperitoneal administration of 2R,3R-trans-dihydroquercetin at concentrations of 150 and 300 mg/kg on the platelet count in the peripheral blood of mice both exposed and not exposed to ionizing radiation at a dose of 7 Gy was studied (Figure 7B). In the group of unirradiated animals (0 Gy), the platelet count in the bloodstream did not change significantly throughout the experiment. Administration of DHQ to intact mice also did not cause any noticeable changes in the platelet count in the bloodstream. After exposure to ionizing radiation, decrease in the platelet count was observed in all groups of animals. The platelet count in mice irradiated at a dose of 7 Gy but not receiving DHQ decreased by slightly less than 90% compared to normal and remained unchanged until death. In the group of irradiated mice receiving DHQ at a concentration of 150 mg/kg, the platelet count in the peripheral blood decreased by 65% by day 7. By the end of the experiment, the platelet count in this group was approximately one-third below normal. In the group of irradiated mice receiving DHQ at a concentration of 300 mg/kg, the platelet count in the peripheral blood was half of normal by day 12. By the end of the experiment, the platelet count in this group was just under 20% below normal. Thus, administration of DHQ significantly reduces the severity of radiation-induced thrombopenia.

The cell counts recorded in each animal in each group are presented in Table 2. At least 2000 polychromatophilic erythrocytes were counted for each animal, and at least 10,000 polychromatophilic erythrocytes were counted for 5 animals. A separate series of experiments was conducted with a smaller number of mice, in which DHQ was administered at a concentration of 300 mg/kg in the absence of irradiation. It was shown that for every 2000 polychromatophilic erythrocytes, there were 8-9 polychromatophilic erythrocytes with micronuclei. Thus, DHQ, even at the highest concentration used, did not induce micronucleus formation in polychromatophilic erythrocytes.

The effect of 2R,3R-trans-dihydroquercetin on the formation of polychromatophilic erythrocytes with micronuclei in the red bone marrow of mice exposed to ionizing radiation at a dose of 1 Gy was studied (Table 3).

It was shown that in intact animals not exposed to ionizing radiation and not receiving DHQ, the red bone marrow contained about half a percent of polychromatophilic erythrocytes with micronuclei. When animals were exposed to ionizing radiation at a dose of 1 Gy, the content of polychromatophilic erythrocytes with micronuclei increased 9-fold compared to the control, to almost 4%. Administration of DHQ to mice 15 min before exposure to ionizing radiation reduced the number of polychromatophilic erythrocytes with micronuclei. This was especially effective with DHQ administration at concentrations of 150 and 300 mg/kg. Thus, with the introduction of DHQ at 150 mg/kg, the number of polychromatophilic erythrocytes with micronuclei decreased by almost 20%. With the introduction of DHQ at 300 mg/kg, the number of polychromatophilic erythrocytes with micronuclei decreased by almost 35%. Thus, DHQ has significant genoprotective potential.

The effect of DHQ on the transcription of stress-response genes in mouse bone marrow cells was studied. Bone marrow was collected 1, 15, and 30 days after irradiation. Data on changes in gene transcription (one day after irradiation with dose of 1 Gy) are presented in Table 4. Gene transcription levels in control, non-irradiated mice that did not receive DHQ did not change significantly over 30 days.

In the group of intact animals treated with DHQ, the level of gene transcription did not change significantly relative to the control (0 Gy) for most of the analyzed genes (13 of 24). A slight increase in the transcription level was observed for the genes encoding HSP90, SOD1, SOD2, Prx5, and XRCC4 (2-4-fold), while a decrease in transcription was recorded for HO-1, Prx1, Prx2, and XRCC5 (2-3-fold), as well as caspase 3 and Prx6 (five-fold), and a sharp decrease in transcription was shown for AP-1 (10-fold). Thus, DHQ injection to intact animals leads to a decrease in the transcription level of genes encoding antioxidant enzymes. This may be due to the fact that DHQ administration to non-irradiated animals caused a change in the ROS balance. A significant decrease in the transcription level of the casp3 gene likely leads to a blockade of processes associated with apoptosis. In the group of mice not receiving DHQ and exposed to ionizing radiation at a dose of 1 Gy, significant changes in the transcription levels of most genes (20 out of 24) were observed 24 h after the start of the experiment. However, the transcription level of four genes changed insignificantly. A significant 20-fold increase in the transcription level was shown for the SOD3 gene. Transcription of the Prx6 gene increased 12-fold. Transcription of the NFkb and Prx2 genes increased 5-6-fold. A slight increase in transcription levels by 2–4 times was found for the XRCC4 and ATR genes. The transcription level decreased by 14 times for the Prx3 gene; by 7 times for the AP-1 and Prx4 genes; and by 4 times for caspase 3. A decrease in the transcription level by 2–3 times was observed for the Prx5, SOD1, SOD2, TNFa, and LigIV genes. In the group of mice treated with DHQ and exposed to ionizing radiation at a dose of 1 Gy, changes in the transcription levels of approximately half of the studied genes (11 of 24) were observed 24 h after the start of the experiment. It was shown that in this group, the transcription level increased 3-fold for the XRCC5, SOD3, and XRCC4 genes and 2-fold for the NFkb, Prx4, and Prx6 genes. As can be seen from Table 4, for irradiated mice treated with DHQ, there is a tendency for the transcriptional activity of genes involved in inflammation and reparation processes to decrease, compared with irradiated mice that did not receive DHQ. For a number of genes, the transcription level in irradiated mice treated with DHQ was close to normal (intact mice). Thus, the introduction of DHQ neutralizes the effect of ionizing radiation and normalizes the transcription level of the studied genes.

## 4. Discussion

It has been shown that DHQ exhibits significant antioxidant properties, reducing the radiation-chemical yield of OH radicals (Figure 2) and hydrogen peroxide (Figure 3) during water radiolysis. It is known that the process of water radiolysis in general looks like this [22]:

H_2_O → ^•^OH + H^•^ + H^+^ + e^−^_aq_ + H_2_ + H_2_O_2_ (1)

During radiolysis in oxygen-free conditions, hydrogen peroxide is formed in the following reaction [23]:

HO^•^ + ^•^OH → H_2_O_2_ (2)

In the presence of dissolved molecular oxygen in water, a hydroperoxide radical is formed during radiolysis:

H^•^ + O_2_^−•^ → HO_2_^•^ (3)

e^−^_aq_ + O_2_ + H^+^ → HO_2_^•^ (4)

Under such conditions, hydrogen peroxide is formed in the following reactions [24]:

H^+^ + HO_2_^•^ → H_2_O_2_ (5)

HO_2_^•^ + HO_2_^•^ → H_2_O_2_ + O_2_ (6)

Thus, the radiation-chemical yield of hydrogen peroxide during radiolysis is affected by the OH-radical, molecular oxygen, hydrated electron, hydrogen atom radical and hydroperoxide radicals [25]. It is known that during radiolysis of pure water, the ratio of formed hydrogen peroxide and hydroxyl radicals is 1:3 [26]. This ratio usually changes insignificantly even with the addition of antioxidants to water. This study showed that in the presence of DHQ at a concentration of 1 mM, the ratio of hydrogen peroxide formed during radiolysis and hydroxyl radicals is approximately 1:2. It follows from this that DHQ molecules are extremely effective against hydroxyl radicals. Probably, one DHQ molecule can neutralize several hydroxyl radicals before inactivation. On the other hand, this may indicate that DHQ molecules are ineffective in preventing reactions with hydrated electrons, hydrogen atom radicals, and hydroperoxide radicals.

It is believed that only 30% of cellular biopolymers are damaged by the direct action of ionizing radiation. Damage to the remaining 70% of biopolymers is due to the action of reactive oxygen species formed during water radiolysis [27]. It should be noted that all reactive oxygen species, except hydrogen peroxide, have short lifetimes [28]. Likely due to its significant antioxidant properties, DHQ prevents radiation-induced damage to 8-oxoguanine (Figure 4), a key marker of oxidative DNA damage and its ambiguous coding properties. Furthermore, DHQ has been shown to prevent the formation of long-lived reactive protein forms (Figure 5). It has previously been shown that exposure of aqueous albumin colloids to ionizing radiation leads to the formation of long-lived reactive protein species with half-lives exceeding 20 h [29]. Such long-lived reactive protein species can damage DNA structure [30]. The genotoxic properties of long-lived reactive protein species in vivo have been well described. In addition to direct interaction with biomolecules, long-lived reactive protein species can participate in the chemical generation of reactive oxygen species [31]. Ascorbic [32], gallic [30], and uric [33] acids, as well as guanosine and inosine [34], can effectively eliminate long-lived active protein forms. We found that the addition of DHQ at a concentration of 1 mM reduces the intensity of the formation of long-lived active protein forms by more than 2.5 times. DHQ has been found to be an effective radioprotective agent (Figure 6). The DRF for DHQ (300 mg/kg) administered intraperitoneally 15 min before irradiation is 1.22. It should be noted that much more effective antiradiation agents exist; however, they all have significant side effects [35]. The absolute record-holder among radioprotectors is WR-2721 (Amifostine). This drug, at a concentration of 500 mg/kg, has a DRF of 2.7 [36]. Amifostine, like other sulfhydryl drugs, is effective at doses close to acute toxic doses [3], and the effective dose of these compounds is quite close to the half-lethal dose. At the same time, DHQ appears quite advantageous when compared with natural non-toxic radioprotective compounds. Currently, only about a dozen such compounds with slightly better radioprotective potential are known. The most effective among natural non-toxic radioprotective compounds are tocotrienol (DRF = 1.29) [37], tocopherol succinate (DRF = 1.28) [38] and orientin (DRF = 1.26) [39]. As can be seen, DHQ, with DRF = 1.22, is quite close to this group of compounds in terms of effectiveness, but it is of completely natural origin. DHQ is obtained by medium-tonnage production, and DHQ is not subject to chemical modification, like, for example, tocopherol succinate.

It is known that exposure to ionizing radiation at doses ranging from 3 to 10 Gy in mice results in a lethal outcome due to the development of hematopoietic syndrome caused by bone marrow cell death [40]. This process is accompanied by depletion of stem cell depots in the bone marrow, leading to changes in the quantitative counts of formed elements in the bloodstream. A decrease in the number of formed elements in the bloodstream leads to weakened immunity and hemorrhages [41]. Intraperitoneal injection of DHQ has been shown to significantly reduce the severity of radiation-induced leukopenia and thrombopenia in experimental animals (Figure 7). These effects are likely due to the genoprotective properties of DHQ (Table 2 and Table 3).

DHQ has been shown to have a significant effect on the transcription of a number of regulatory genes (Table 4). When analyzing the effect of DHQ on intact organisms (the “0 Gy + DHQ” group), it was established that administration of the drug to non-irradiated animals is not a physiologically neutral event. Changes in transcriptional activity were recorded for 11 of the 24 genes studied, with the direction of the changes being predominantly suppressive. The most pronounced decrease in expression was observed for the genes AP-1 (10-fold); casp3 and Prx6 (5-fold); and HO-1, Prx1, Prx2 and XRCC5 (2-3-fold). The obtained results can be interpreted as a consequence of a decrease in the basal level of reactive oxygen species (ROS) due to the direct antioxidant activity of DHQ. Under conditions of reduced oxidative background, the cell reduces the expression of endogenous antioxidant enzymes, which is a physiologically adequate compensatory response. However, as some authors rightly note, the regulatory function under conditions of oxidative stress can be performed by both DHQ itself and its oxidized form [42]. The chemical reactivity of the oxidized antioxidant is crucial because oxidized flavonoids, being electrophiles, are capable of transferring disordered energy to other antioxidant molecules, such as glutathione and ascorbate, or directly to redox switches [43]. This, in turn, leads to the activation of redox-sensitive signaling cascades and induces an adaptive cellular response. Thus, the observed decrease in the expression of endogenous antioxidant genes in intact animals may reflect not only a compensatory response to a decrease in the ROS pool but also a consequence of the “bioactivation” of DHQ to the corresponding electrophilic quinones, which redirect cellular metabolism toward an adaptive phenotype without the need for overexpression of protective genes.

The simultaneous suppression of transcription of the caspase 3 gene and the transcription factor AP-1 indicates a cytoprotective effect of DHQ aimed at inhibiting constitutive apoptosis and basal inflammatory signaling. Along with suppressive effects, intact animals receiving DHQ showed a moderate increase in the expression of the HSP90, SOD1, SOD2, Prx5, and XRCC4 genes (2-4-fold), which may reflect a mild preactivation of the stress response and DNA repair systems, creating a state of “preadaptation” (preconditioning effect) to potential damaging effects.

Exposure to 1 Gy of ionizing radiation in animals not receiving DHQ induced a pronounced transcriptional imbalance, affecting 20 of the 24 genes studied. The pattern of changes was consistent with the classic pattern of acute radiation stress response. The most dramatic changes were recorded for the SOD3 gene, whose transcription level increased 20-fold relative to control levels. Given that SOD3 encodes the extracellular form of superoxide dismutase, this observation reflects the systemic nature of oxidative damage and the mobilization of the extracellular antioxidant defense compartment. Significant transcriptional activation was also detected for the Prx6 genes (12-fold increase), NF-kB and Prx2 (5–6-fold increase), and XRCC4 and ATR (2-4-fold increase). Along with the activation of defense systems, profound suppression of the expression of a number of genes was observed: the most pronounced decreases were recorded for Prx3 (14-fold); AP-1 and Prx4 (7-fold); casp3 (4-fold); and Prx5, SOD1, SOD2, TNFα, and LigIV (2–3-fold). Particularly noteworthy is the sharp suppression of transcription of the Prx3 gene, which encodes the mitochondrial form of peroxiredoxin. Mitochondria are a critical target of radiation damage, and suppression of the expression of a key mitochondrial antioxidant enzyme creates the preconditions for the development of energy deficiency, increased mitochondrial ROS production, and the initiation of apoptosis via the intrinsic pathway. Reduced expression of DNA repair genes (LigIV) coupled with activation of sensor kinases (ATR) indicates an imbalance in the repair response, which may contribute to the fixation of DNA damage and genomic instability.

A fundamentally different picture was observed in the group of animals receiving DHQ 15 min before irradiation. DHQ premedication significantly modified the transcriptional response to radiation exposure: expression changes affected only 11 of 24 genes, and the amplitude of these changes was significantly lower than in the irradiated group without protection. The most important result was the prevention of the radiation-induced decline in Prx3 gene expression: in the “1 Gy + DHQ” group, the transcription level of this gene practically corresponded to control values and was 16 times higher than in the group of irradiated animals without DHQ. A similar normalization was observed for the Prx4 gene. Given the key role of peroxiredoxins in maintaining redox homeostasis of mitochondria and the endoplasmic reticulum, this effect can be considered one of the central mechanisms of DHQ’s radioprotective action. Restoring the expression of mitochondrial antioxidant systems appears to preserve mitochondrial functional activity and prevent the initiation of apoptotic cascades. In the group of irradiated animals receiving DHQ, moderate activation of DNA repair genes was observed: XRCC4 and XRCC5 expression increased threefold, while ATR expression increased fourfold. Importantly, unlike the group without DHQ, where ATR activation was accompanied by LigIV suppression, DHQ treatment resulted in balanced activation of both sensory and effector components of the DNA double-strand break repair system. This suggests that DHQ promotes a coordinated and physiologically appropriate repair response, minimizing the risk of damage fixation and mutations. Analysis of apoptotic and inflammatory gene expression revealed a pronounced normalizing effect of DHQ. While a fourfold decrease in caspase 3 expression was observed in the irradiated group without DHQ, likely reflecting post-transcriptional blockade of protein synthesis in cells entering apoptosis or the elimination of cells with high levels of this gene expression, casp3 transcription levels in the DHQ group corresponded to control values. Similar normalization was recorded for the NF-kB and AP-1 genes: moderate activation of NF-kB (2-fold) and the absence of AP-1 suppression indicate the maintenance of physiological levels of proinflammatory signaling without pathological hyperactivation or depletion. The behavior of the SOD3 gene deserves special discussion. In all experimental groups, including intact animals receiving DHQ, SOD3 transcription levels exceeded control values. In the 1 Gy + DHQ group, gene expression increased 3-fold, while in the group without DHQ, it increased 20-fold. Thus, DHQ does not completely normalize SOD3 expression, but it significantly limits the amplitude of its induction. Given that SOD3 is a secreted protein and the main extracellular scavenger of superoxide anion, the persistence of its elevated expression 24 h after irradiation may reflect the incomplete elimination of radiolytic products and the persistence of residual oxidative stress in the extracellular space. An alternative explanation may be the relatively slow normalization of this transcriptional response compared to intracellular antioxidant systems. The p53 gene demonstrated remarkable stability of expression in all studied groups. Given the central role of p53 in the response to DNA damage, the lack of significant changes in its transcription 24 h after irradiation may be due to the fact that the peak activation of p53-dependent transcription occurs earlier (2–6 h after irradiation). Furthermore, it is well known that regulation of the p53-mediated response occurs primarily at the post-regulatory level—through phosphorylation, acetylation, and protein stabilization—while transcription of the p53 gene itself varies within relatively narrow limits. These data do not exclude the possibility that DHQ modulate p53 activity at the protein level or its ability to transactivate target genes.

**Figure 8:**
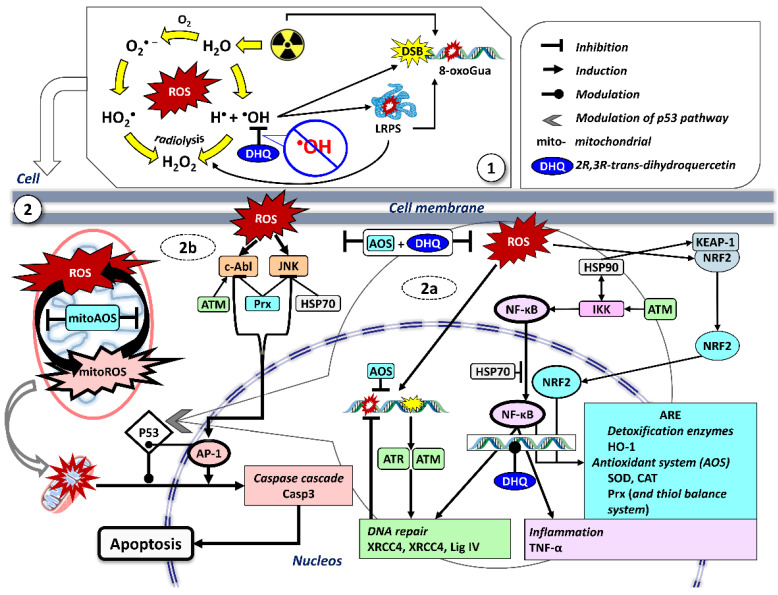
The role of 2R,3R-trans-dihydroquercetin (DHQ) in the response to ionizing radiation at the molecular and cellular level. 1– Neutralization of the hydroxyl radical (^•^OH) formed during radiolysis of the aqueous medium and subsequent reactions of reactive oxygen species (ROS) formation, as well as the processes of long-lived reactive protein species (LPRS) formation and oxidative DNA damage induced by it (using 8-oxoguanine (8-oxoGua) as an example). 2—Participation of DHQ in cellular signaling after irradiation. The main processes and their key participants are presented, the expression of most of which is subject to the modulating effect of DHQ. 2a—DNA damage and the NF-κB pathway. ROS cause DNA damage, which leads to the activation of sensor kinases (ATM (ataxia-telangiectasia mutated protein), ATR (ataxia-telangiectasia protein and Rad3-related protein)). ATM/ATR phosphorylate proteins (p53 and checkpoint kinases), leading to cell cycle arrest and activation of DNA repair pathways. Double-strand breaks are primarily repaired through non-homologous end joining, which requires the XRCC4, XRCC5, and DNA ligase IV (LigIV) genes. ATM also phosphorylates and activates the IKK complex. IKK phosphorylates the NF-κB inhibitor, leading to its degradation and the release of NF-κB, which translocates to the nucleus. In the nucleus, NF-κB induces the expression of genes responsible for survival (DNA repair) and inflammation, including cytokines and chemokines (TNF-α-tumor necrosis factor alpha), which enhance the inflammatory response. At the same time, ROS disrupt the binding of NRF2 (nuclear factor erythroid 2-related factor 2) to Keap1 (Kelch-like ECH-associated protein 1), causing its nuclear translocation. NF-κB and NRF2 jointly regulate ARE (antioxidant response element: HO-1 (heme oxygenase-1), which destroys the prooxidant heme; ROS scavengers—superoxide dismutase (SOD), catalase (CAT); peroxiredoxins (Prx)—multifunctional peroxidases with signaling and chaperone functions, involved in thiol balance; etc.. As a result, DNA damage can be neutralized. Critical DNA damage activates p53 and triggers apoptosis, mainly through mitochondrial pathway and activation of the caspase cascade (Casp3 is the key effector caspase-3). 2b—ROS activate the transcription factor AP-1 via protein kinases JNK (c-Jun N-terminal kinases) and c-Abl (tyrosine kinase Abl1). The JNK/AP-1 signaling pathway also stimulates apoptosis, and ATM-mediated phosphorylation of c-Abl (tyrosine kinase Abl1) regulates it. Heat shock proteins (HSP70 and HSP90) have additional regulatory functions.

Summarizing the obtained results, the following model for the radioprotective effect of 2R,3R-trans-dihydroquercetin can be proposed. The drug, administered prior to irradiation, restores the redox status in bone marrow cells through direct inactivation of reactive oxygen species and, possibly, chelation of transition metal ions. Upon subsequent exposure to ionizing radiation, the primary radiation-chemical damage caused by water radiolysis is partially compensated by the antioxidant present in the tissue. As a result, the intensity of oxidative stress and, accordingly, the amplitude of signals activating redox-sensitive transcription factors are reduced. This allows the cell to generate an adequate, but not excessive, transcriptional response, characterized by moderate activation of protective systems while maintaining their functional reserve and preventing exhaustion. A critical component of this response is the preservation of mitochondrial peroxiredoxin expression, which ensures the maintenance of energy metabolism and inhibition of the mitochondrial apoptotic pathway. The data presented in the present study on the ability of DHQ to normalize the transcriptional profile of bone marrow cells under radiation exposure open up prospects for the clinical use of this compound. Of particular interest is the potential use of DHQ in radiation therapy of oncological diseases. As shown in recent studies, DHQ exhibits antitumor activity against various malignancies due to the regulation of multiple signaling pathways, including Wnt/β-catenin, phosphoinositide 3-kinase (PI3K)/protein kinase B (Akt), mammalian target of rapamycin (mTOR), transforming growth factor beta (TGF-β) and NF-κB [44]. The antitumor mechanisms of DHQ include inhibition of cell proliferation, invasion and migration; induction of cell cycle arrest; activation of autophagy and apoptosis; epigenetic modification; suppression of epithelial-mesenchymal transition; increased effectiveness of chemotherapy and enhancement of immune function. Thus, DHQ possesses a unique combination of properties: on the one hand, it protects normal tissues from the damaging effects of ionizing radiation; on the other, it potentiates the antitumor effect and can act as an independent cytostatic agent. Of particular note is DHQ’s ability to improve regeneration, reduce inflammation, and prevent skin carcinogenesis [45]. Radiation-induced skin damage is one of the most common complications of radiation therapy for malignant tumors, affecting up to 95% of patients [46]. In this context, our discovery of normalization of gene expression of proinflammatory cytokines and antioxidant enzymes in bone marrow cells under the influence of DHQ suggests that similar mechanisms may be at work in other tissues, including skin and mucous membranes, located within the irradiated zone. The ability of DHQ to limit NF-kB hyperactivation and prevent AP-1 suppression demonstrated in this study correlates with the anti-inflammatory effects described in dermatological studies. Figure 8 presents a hypothetical schematic of possible scenarios following exposure to ionizing radiation and the possible role of DHQ in them. The most likely key targets for DHQ signaling are the NF-kB and AP-1 genes.

## 5. Conclusions

Thus, 2R,3R-trans-dihydroquercetin exhibits properties of an effective antioxidant and radioprotector, the mechanism of action of which is fundamentally different from classical stress response inducers (such as bacterial polysaccharides or synthetic cytokines) and is based on the normalization of the target cell transcriptional profile, rather than its hyperstimulation. The ability of DHQ to restore the expression of antioxidant defense, DNA repair, and apoptotic genes to physiological levels under radiation exposure allows this compound to be considered a promising pharmacological agent for the correction of radiation damage to normal tissues that occurs during radiotherapy for malignant neoplasms. Further research should be aimed at verifying the observed effects at the protein level, assessing the functional state of mitochondria, studying the role of oxidized DHQ metabolites in the implementation of its biological effects, and investigating the long-term consequences of DHQ use in the post-radiation period.

## Figures and Tables

**Table 1:** Oligonucleotides used for qRT-PCR.

Genes	GenBank Accsession #	Oligonucleotide 5′-3′	Amplicon Size, bp
*Actb*	NM_007393.4	CCTTCCTTCTTGGGTATGGAATCC	115
		CACCAGACAGCACTGTGTTGGCA	
*p53*	NM_001127233	CGAAGACTGGATGACTGCCA	137
		CGTCCATGCAGTGAGGTGAT	
*HO-1*	NM_010442	GATAGAGCGCAACAAGCAGAA	111
		CAGTGAGGCCCATACCAGAAG	
*HSP70*	NM_024172	CACGTTCGACGTGTCCATCCTG	105
		ACCAGCCGGTTGTCGAAGTCCT	
*HSP90*	NM_011631	GTCCGCCGTGTGTTCATCAT	168
		GCACTTCTTGACGATGTTCTTGC	
*NFkb*	NM_008689	CCACGCTCAGCTTGTGAGGGAT	106
		GGCCAAGTGCAGAGGTGTCTGAT	
*Prx1*	NM_011034	AATGCAAAAATTGGGTATCCTGC	149
		CGTGGGACACACAAAAGTAAAGT	
*Prx2*	NM_011563	CACCTGGCGTGGATCAATACC	138
		GACCCCTGTAAGCAATGCCC	
*Prx3*	NM_007452	GGTTGCTCGTCATGCAAGTG	99
		CCACAGTATGTCTGTCAAACAGG	
*Prx4*	NM_016764	CTCAAACTGACTGACTATCGTGG	101
		CGATCCCCAAAAGCGATGATTTC	
*Prx5*	NM_012021	GGCTGTTCTAAGACCCACCTG	154
		GGAGCCGAACCTTGCCTTC	
*Prx6*	NM_007453	TAAGGACAGGGACATTTCCATCC	145
		CCGTGGAGTTAGGGTAGAGGA	
*Xrcc4*	NM_028012	GAGACACCGAATGCAGAAGA	121
		GGTGCTCTCCTCTTTCAAGG	
*Catalase*	NM_009804	AGCGACCAGATGAAGCAGTG	181
		TCCGCTCTCTGTCAAAGTGTG	
*SOD1*	NM_011434	AACCAGTTGTGTTGTCAGGAC	139
		CCACCATGTTTCTTAGAGTGAGG	
*SOD2*	NM_013671	GCGGTCGTGTAAACCTCAT	240
		CCAGAGCCTCGTGGTACTTC	
*SOD3*	NM_011435	CTGAGGACTTCCCAGTGAC	195
		GGTGAGGGTGTCAGAGTGT	
*AP-1*	NM_010591	CACGGAGAAGAAGCTCACAA	126
		ACTTGTTACCGGTCCTCTGG	
*ATR*	NM_019864	GAATGGGTGAACAATACTGCTGG	107
		TTTGGTAGCATACACTGGCGA	
*ATM*	NM_007499.2	TACATCCTTGGACTTGGCGAC	88
		GCCACTCCCAGGTCTATGTG	
*Xrcc5*	NM_009533	GAAGAACAGCGCTTCAACAG	92
		TCCTGAACAACAATTTCCCA	
*LigIV*	NM_176953	ATGGCTTCCTCACAAACTTCAC	103
		TTTCTGCACGGTCTTTACCTTT	
*Caspase 3*	NM_009810	AAGGAGCAGCTTTGTGTGTG	145
		GAAGAGTTTCGGCTTTCCAG	
*TNFa*	NM_013693	ATGAGAAGTTCCCAAATGGC	125
		CTCCACTTGGTGGTTTGCTA	
*XRCC4*	NM_028012	GAGACACCGAATGCAGAAGA	121
		GGTGCTCTCCTCTTTCAAGG	
*XRCC5*	NM_009533	GAAGAACAGCGCTTCAACAG	92
		TCCTGAACAACAATTTCCCA	

Calculated Tm for all primers 61–63 °C. Real-time PCR was performed at Tm = 62 °C. After PCR was performed, amplicons were melted (from 60 to 90 °C), and after chilling, gel electrophoresis was performed in 2% agarose gel.

**Table 2:** Number of cells recorded in each animal of each group.

No.	0 Gy		1 Gy							
			DHQ, mg/kg							
			0		30		150		300	
1	2000	(10)	2002	(82)	2007	(73)	2053	(74)	2000	(46)
2	2000	(8)	2114	(88)	2024	(68)	2039	(58)	2001	(70)
3	2061	(9)	2009	(78)	2081	(77)	2004	(61)	2008	(52)
4	2039	(8)	2137	(85)	2031	(82)	2041	(67)	2033	(43)
5	2016	(7)	2000	(71)	2016	(79)	2010	(67)	2011	(50)
∑	10,116	(42)	10,262	(404)	10,159	(379)	10,147	(327)	10,053	(261)

No. is the animal’s serial number. For each experimental group, the total number of counted cells is indicated on the left for each animal; the number of counted polychromatophilic erythrocytes containing micronuclei is indicated in parentheses on the right. ∑—total for all animals in the group.

**Table 3:** The effect of 2R,3R-trans-dihydroquercetin on the formation of polychromatophilic erythrocytes with micronuclei in the red bone marrow of mice exposed to ionizing radiation at a dose of 1 Gy.

No	Group	PCE with MN, %	*P* (According to Student’s Criterion)				
			No1	No2	No3	No4	No5
1	0 Gy	0.42 ± 0.03		<0.01	<0.01	<0.01	<0.01
2	1 Gy	3.93 ± 0.11	<0.01		0.24	<0.01	<0.01
3	1 Gy + DHQ 30 mg/kg	3.73 ± 0.12	<0.01	0.24		0.02	<0.01
4	1 Gy + DHQ 150 mg/kg	3.22 ± 0.13	<0.01	<0.01	0.02		0.05
5	1 Gy + DHQ 300 mg/kg	2.60 ± 0.24	<0.01	<0.01	<0.01	0.05	

**Table 4:** Effect of 300 μg/g 2R,3R-trans-dihydroquercetin administered intraperitoneally to mice 15 min before whole-body irradiation with dose of 1 Gy on the number of mRNA copies 24 h after irradiation.

Genes	Relative Gene Expression			
	0 Gy		1 Gy	
	-	DHQ	-	DHQ
*p53*	4.5 × 10^−5^	4.2 × 10^−5^	2.1 × 10^−5^	5.0 × 10^−5^
*HO-1*	8.5 × 10^−3^	2.9 × 10^−3^	9.5 × 10^−3^	5.8 × 10^−3^
*HSP70*	2.0 × 10^−3^	5.4 × 10^−4^	8.6 × 10^−4^	5.1 × 10^−4^
*HSP90*	10 × 10^−2^	2.8 × 10^−1^	6.1 × 10^−2^	8.1 × 10^−2^
*NFkb*	1.7 × 10^−4^	2.8 × 10^−4^	9.3 × 10^−4^	3.7 × 10^−4^
*Prx1*	5.2 × 10^−2^	2.7 × 10^−2^	5.7 × 10^−2^	9.1 × 10^−2^
*Prx2*	12.0 × 10^−2^	9.5 × 10^−2^	80.3 × 10^−2^	9.7 × 10^−2^
*Prx3*	5.7 × 10^−3^	6.5 × 10^−3^	3.9 × 10^−4^	6.3 × 10^−3^
*Prx4*	1.2 × 10^−3^	9.0 × 10^−3^	1.6 × 10^−4^	2.4 × 10^−3^
*Prx5*	6.1 × 10^−2^	11.1 × 10^−2^	2.1 × 10^−2^	4.7 × 10^−2^
*Prx6*	3.4 × 10^−3^	7.9 × 10^−4^	4.4 × 10^−2^	8.7 × 10^−3^
*Cat*	2.2 × 10^−3^	2.2 × 10^−3^	0.7 × 10^−3^	1.6 × 10^−3^
*SOD1*	4.1 × 10^−2^	8.2 × 10^−2^	1.2 × 10^−2^	1.8 × 10^−2^
*SOD2*	2.6 × 10^−6^	5.3 × 10^−5^	1.4 × 10^−6^	2.8 × 10^−5^
*SOD3*	1.3 × 10^−3^	1.5 × 10^−3^	26.1 × 10^−3^	5.7 × 10^−3^
*Ap-1*	10 × 10^−3^	1.0 × 10^−3^	1.3 × 10^−3^	1.1 × 10^−3^
*ATR*	2.8 × 10^−4^	1.4 × 10^−4^	6.4 × 10^−4^	2.5 × 10^−3^
*ATM*	18 × 10^−3^	5.7 × 10^−3^	6.9 × 10^−3^	9.5 × 10^−3^
*LigIV*	6.5 × 10^−3^	6.0 × 10^−3^	2.4 × 10^−3^	5.1 × 10^−3^
*casp3*	2.2 × 10^−2^	0.5 × 10^−2^	0.5 × 10^−2^	3.0 × 10^−2^
*TNFa*	1.7 × 10^−3^	1.5 × 10^−4^	0.8 × 10^−4^	1.3 × 10^−4^
*Xrcc4*	1.8 × 10^−3^	4.1 × 10^−3^	8.6 × 10^−3^	5.2 × 10^−3^
*Xrcc5*	6.0 × 10^−3^	3.4 × 10^−3^	9.6 × 10^−3^	1.9 × 10^−2^

## Data Availability

The datasets presented in this article are not readily available because [Order of the Director of the Institute of Theoretical and Experimental Biophysics of the Russian Academy of Sciences No. 7/26]. Requests to access the datasets should be directed to 3 Institutskaya st., Pushchino, Moscow Region, 142290 Russia.

## Abbreviations

The following abbreviations are used in this manuscript:

DHQ	Dihydroquercetin
DRF	Dose Reduction Factor
ROS	Reactive Oxygen Species
8-oxoGua	8-oxoguanine
